# MACE: Automated Assessment of Stereochemistry of Transition Metal Complexes and Its Applications in Computational Catalysis

**DOI:** 10.1021/acs.jctc.3c01313

**Published:** 2024-02-16

**Authors:** Ivan Yu. Chernyshov, Evgeny A. Pidko

**Affiliations:** Inorganic Systems Engineering, Department of Chemical Engineering, Faculty of Applied Sciences, Delft University of Technology, Van der Maasweg 9, 2629 HZ, Delft, The Netherlands

## Abstract

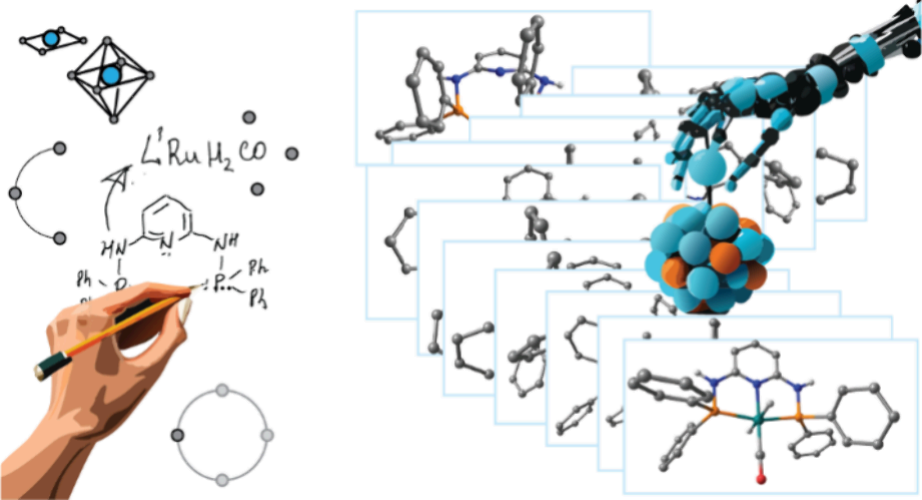

Computational chemistry pipelines typically commence with geometry generation, well-established for organic compounds but presenting a considerable challenge for transition metal complexes. This paper introduces MACE, an automated computational workflow for converting chemist SMILES/MOL representations of the ligands and the metal center to 3D coordinates for all feasible stereochemical configurations for mononuclear octahedral and square planar complexes directly suitable for quantum chemical computations and implementation in high-throughput computational chemistry workflows. The workflow is validated through a structural screening of a data set of transition metal complexes extracted from the Cambridge Structural Database. To further illustrate the power and capabilities of MACE, we present the results of a model DFT study on the hemilability of pincer ligands in Ru, Fe, and Mn complexes, which highlights the utility of the workflow for both focused mechanistic studies and larger-scale high-throughput pipelines.

## Introduction

Computational exploration of transition metal chemical space is, or at least is expected to be, a key part of the rational development of new catalysts^1−4^ and advanced functional materials with promising optical, sensing, and magnetic properties.^5−7^ Progress in development of computational and data-driven tools for transition metal complexes (TMCs) has considerably facilitated such research.^8−11^ Special note must be made of the recently introduced xTB software supporting molecular mechanics and semiempirical methods, which provide robust and powerful toolsets for quick geometry preoptimization of TMCs,^12^ and fast highly accurate post-Hartree–Fock methods such as DPLNO-CCSD(T), allowing the complex electronic structure problems of the TMC chemistry that are inaccessible to conventional DFT calculations to be rationalized.^13,14^ The transition toward automated in silico TMC exploration and analysis has been greatly facilitated by the introduction of reliable three-dimensional (3D) structure generation tools, which partially or completely automate the construction of starting molecular models.^15−18^

Despite the different logic of the underlying algorithms, the main functionality of these tools is to generate 3D atomic coordinates of the most stable configuration(s) for a given metal complex, which is usually described as a set of a central ion, its geometry, and ligands’ 1D/2D representations (mainly SMILES or MOL-file). This represents a powerful approach for material design, where the most stable configuration almost always determines the functional characteristics. However, in catalysis, where metastable or minor configurations may often determine the overall performance, an insight into the ensemble characteristics or, at least, detailed information about the properties and relative stabilities of all possible stereoisomers of a TMC is necessary.

Although most currently available 3D generation tools support the description of the stereochemistry of TMCs,^15−18^ there is no straightforward way to use them for the full stereochemistry analysis, which in addition to the generation of all possible stereoisomers requires an algorithm comparing stereoisomers to filter out identical configurations, and an additional functionality to filter out “impossible” configurations, corresponding to high-energy or nonlocalizable systems.

We address this problem with the introduction of the MACE package, which is designed for the generation of 3D atomic coordinates for all possible stereoisomers of a given octahedral or square-planar mononuclear complex. Unlike molAssembler,^16^ which solves the problem of the stereochemistry of transition metal compounds in a general way, we limited ourselves to mononuclear complexes, where all stereoisomers can be enumerated via atomic labels on donor atoms and compared using unique SMILES (USMILES). RDKit was used as an internal engine, which was supplemented with the logic for stereomer search and generation of 3D coordinates with consideration of the central atom’s stereochemistry. The final product is a python library with a CLI interface that was successfully tested as a geometry generator in the routine work of our group.^19−21^

Besides the utility as a starting point of in silico catalyst screening campaigns, MACE is designed to be compatible with automated experimental catalyst evaluation workflows, as a robust tool to automate accompanying DFT calculations of computational descriptors based solely on the chemically intuitive input from nonexperts in computational chemistry.

The paper is organized as follows. We start with a description of the underlying algorithm of MACE. Next, we present the process and results of validation of MACE on an extended experimental structural data set of TMCs. The results section is followed by the presentation of two use cases exploring the hemilability of transition metal pincer complexes.

## Methods

The Cambridge Structure Database (CSD) version 5.42 with updates until May 2021 was inspected using the ConQuest program.^22^ All queries were limited to crystal structures that contained 3D coordinates and had no errors. Entries containing 4-coordinated Pd and Pt and 6-coordinated Mn and Ru were extracted in the SMILES format. The subsequent extraction of multidentate ligands was carried out using standard chemoinformatic methods using the RDKit package. For implementation details on the validation pipeline and all of the initial, intermediate, and final data we refer to the “performance” section of the 4TU.ResearchData data set containing MACE’s source code^23^ or of the GitHub repository.^24^

Similar to our previous works on transition metal pincer complexes,^19−21^ density functional theory (DFT) calculations were performed with the PBE0 (also denoted as PBE1PBE and PBEh)^25^ hybrid exchange-correlation functional using the Gaussian 16, revision C.01, program.^26^ Our combined computational and experimental studies on related chemical systems as well as earlier extensive prior benchmarking studies^27,28^ confirm the high accuracy of this method. The double-zeta quality basis set was used in all calculations, namely, def2-SVP for the systems with the PN^3^P ligand and 6-31G(d,p)/SDD(Ru) for all other systems. 3D atomic coordinates for all complexes in this study were generated via Python scripts using the epic-mace package (PyPI alias of MACE).^24,29^ All operations with data, including processing of Gaussian input- and output-files, analysis of geometrical parameters of generated complexes, and preparation of test ligands, were carried out using Python and the RDKit library. The corresponding xyz-files and xlsx-tables containing relative electronic energies can be found in the Supporting Information and the accompanying TU.ResearchData data set.^30^

## Software Description

**Figure 1:**
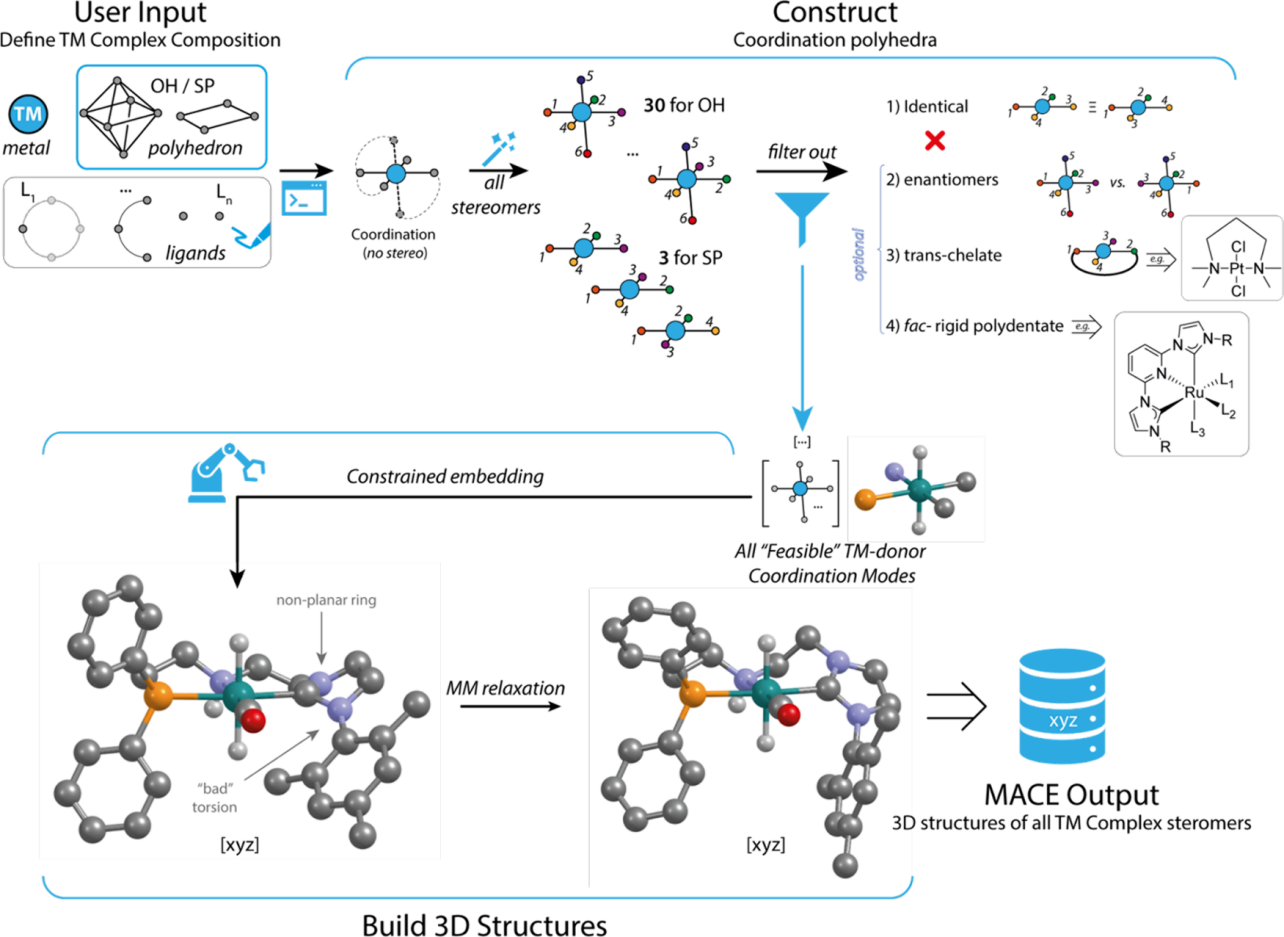
Flowchart of the MACE package.

The general overview of the MACE pipeline is schematically presented in Figure 1 and will be discussed in the following text in detail. MACE targets the generation of all possible coordination environments in the form of atomic coordinates for a given set of central metals and ligands. The procedure starts with the transformation of an input metal–ligand chemical system defined by a central ion, its coordination geometry, and SMILES-encoded ligands into a set of all possible coordination complex stereoisomers. First, structures covering all possible spatial arrangements of the donor atoms around the metal center are generated. Fundamentally, there are 30 possible arrangements for an octahedral environment (OH) and only 3 for square-planar (SP) geometries (see the “Counting number of isomers for square planar and octahedral stereocenters” section in the SI).

**Figure 2:**
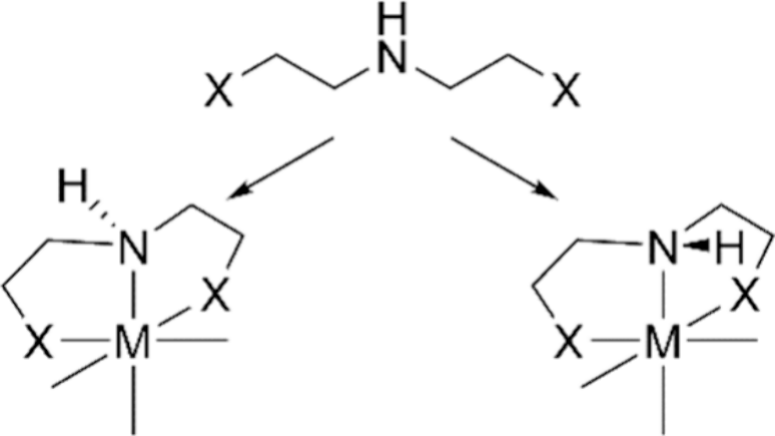
Geometrical isomerism induced by stereoconfiguration of donor secondary amine atom exemplified on the model XNX ligand.

The number of possible configurations may increase due to chirality on the coordinated donor atoms, e.g., asymmetric amines or phosphines (Figure 2). The spatial arrangement of donor atoms is encoded via atomic map numbers and/or isotope labels in the SMILES definition of the ligands.

Next, for all constructed coordination polyhedra, the MACE algorithm filters out identical configurations. To compare the two stereoisomers, we use Unique SMILES generated by the RDKit canonicalization algorithm, which works ideally for comparing configurations containing exclusively tetrahedral stereocenters.^31^ To take into account the stereochemistry of the metal center, which is determined by the atomic labels of donor atoms, we generate a set of all possible USMILES corresponding to a given configuration and obtained by various permutations of the atomic labels of the donor atoms. For octahedral and square planar geometries, there are only 24 and 8 such permutations, respectively. Within this approach, two configurations are equivalent if their corresponding USMILES sets have at least one element in common. The same procedure is used to screen out enantiomers, which can be useful if the generated complexes are expected to be further treated in an achiral environment.

The final filtration step is the identification of high-energy and potentially nonlocalizing configurations via search for specific substructures, namely, (1) bidentate ligands with trans-oriented donor atoms, resulting in formation of constrained small-sized ring containing a linear three-atomic fragment, and (2) *fac*-oriented three-dentate rigid ligands like terpyridines. In most cases applying these options eliminates undesired configurations and thus saves computational time; however, for some ligand–metal systems chemical expert knowledge behind those rules may fail by ruling out low-energy isomers (∼10 kJ/mol). Therefore, in the case of investigating a new system, it is usually advisory to ignore those filters.

After all unique stereoisomers of donor-metal arrangements are identified, MACE proceeds with building the ligand environment in 3D atomic coordinates. The built-in RDKit distance geometry method is employed for this purpose.^32^ To achieve the correct stereoconfiguration, a Bounds Matrix containing minimum and maximum distances between atoms is populated with accurate data on the distances between donor atoms and the central atom. Subsequently, after structure generation, the correct spatial arrangement of the donor atoms is verified. Following this verification, the structure undergoes relaxation using the Universal Force Field (UFF), supplemented with missing parameters for bonds, angles, and torsional angles that include dative bonds. This is an intrinsic limitation of the current implementation, although higher levels of theory are normally used to optimize the generated structures. The generated stereoisomers can be stored in the XYZ format with the comment field containing information about the molecular structure, facilitating their reload into MACE without information loss.

MACE can also be used to generate complexes with specific stereochemistry. For this purpose, one should correctly enumerate donor atoms with atomic map numbers in the input section and generate 3D coordinates for the resulting complex without the interim stereoisomer search. This approach can also be used to construct linear or square-pyramidal structures as a special case of octahedra with 2 or 5 defined donor atoms.

Among the critical limitations of the current MACE workflow is the lack of support for π-bonding and polyhaptic ligands. Additionally, challenges may arise with axial symmetry, as RDKit does not provide support for it.

The MACE software has two main ways to use it, namely, Command Line Interface (CLI) and Python scripting. The former is most convenient when isomers are generated for several complexes. The representative examples can be found in the “examples” section of the 4TU.ResearchData data set containing MACE’s source code^23^ or of the GitHub repository.^24^

Python scripting becomes particularly valuable for addressing high-throughput tasks, as straightforward scripts can facilitate the organization of a structured file system for the generated structures. This includes folder names and files describing the peculiarities of the ligand structures. An illustrative example of such usage is presented in more detail below. For the code snippets demonstrating all possible usage scenarios of the epic-mace package we refer to the IPython notebooks provided in the SI or to the “Package cookbook” section of the online documentation.^29^

## Validation

**Figure 3:**
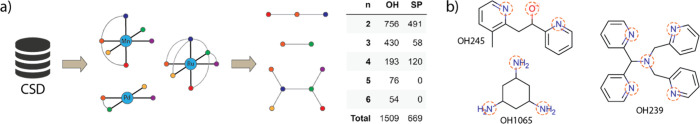
(a) Schematic illustration of the extraction of multidentate ligands from CSD and generating statistics on the denticity of the extracted ligands (n). (b) A representative set of ligands for which MACE is partially or completely unable to generate 3D atomic coordinates; red dotted circles denote donor atoms.

MACE was tested on complexes formed by multidentate ligands (n = 2...6) extracted from the Cambridge Structural Database^22^ from experimental crystal structures of octahedral complexes of Ru and Mn and square-planar complexes of Pd and Pt (Figure 3a). For this purpose, we selected all unique organometallic entries from CSD, from which we extracted all octahedral and square planar mononuclear nonpolymeric complexes of Ru/Mn/Pd/Pt. The obtained structures were then split into the central metal and ligands. Because the spatial arrangement of monodentate ligands is trivial, only the ligands with denticities of 2 and greater were included in the test set, composed in total of 1509 and 669 entries for octahedral and square planar geometries, respectively. Statistical characterization of these two ligand data sets including information on the distributions of the donor atoms, denticity, and connectivity is provided in SI (Table S2, Figures S2–S5).

The extracted ligands were used to generate 3D atomic coordinates for all found stereoisomers of octahedral Ru(CO)_6–n_L and square-planar Pd(CO)_4–n_L complexes, where n is the denticity of the ligand L. The generated 3D structures for the coordination complexes were both automatically and manually assessed for the correct geometry of the central metal and chemically reasonable arrangement of the donor atoms, mainly with respect to the planarity of the sp^2^ and the distortion degree of the tetrahedron of the sp^3^ atoms.

MACE failed to generate 3D coordinates for only 7 complexes out of more than 2000, and in six cases, it was only one of two localized stereoisomers (close structural analogues of OH245 and OH239, Figure 3b). Close examination revealed that in those stereoisomers donor atoms were arranged around the central ion in an “impossible” fashion requiring entanglement of some linkers. This is a flaw in the algorithm for generating stereoisomers, more precisely in the filtering rules for “unlikely” configurations. However, this problem should not affect the results of any computational pipelines, since the absence of the generated conformation most likely indicates its exceptionally high energy and that it can be neglected.

The only ligand for which MACE did not generate any conformer has a surprisingly simple structure (OH1065, Figure 3b). Close examination of the issue allowed us to understand that its origin lies within the RDKit implementation of the distance geometry method, which sometimes fails for highly symmetrical systems.

## Applications

### Conformational and Hemilability Explorations in Organometallic Chemistry

To illustrate the practical capabilities and potential of the MACE methodology, we considered a model system of Ru-PN_3_P pincer complex,^33^ for which the structure has been resolved and which has earlier been used as a catalyst for CO_2_ and bicarbonate hydrogenation.^34,35^ X-ray crystal structure gives an unambiguous solution to the structural problem of coordination complexes by providing a global minimum configuration in the solid state.

The presence of coordinating solvents, reactants, and reaction intermediates under the catalytic conditions may open paths for the ligand exchange and fluxionality, resulting in the formation of alternative configurations, whose structures are not known a priori. Herein, we employ MACE to explore ligand fluxionality of the rigid and highly stable RuPN_3_P complex in the presence of a model representative coordinating solvent acetonitrile (MeCN). The MACE workflow was initiated by specifying with extended SMILEs the building block of the complex, namely, the pincer ligand, the metal center, and auxiliary ligands (two hydrides and a carbonyl).

**Figure 4:**
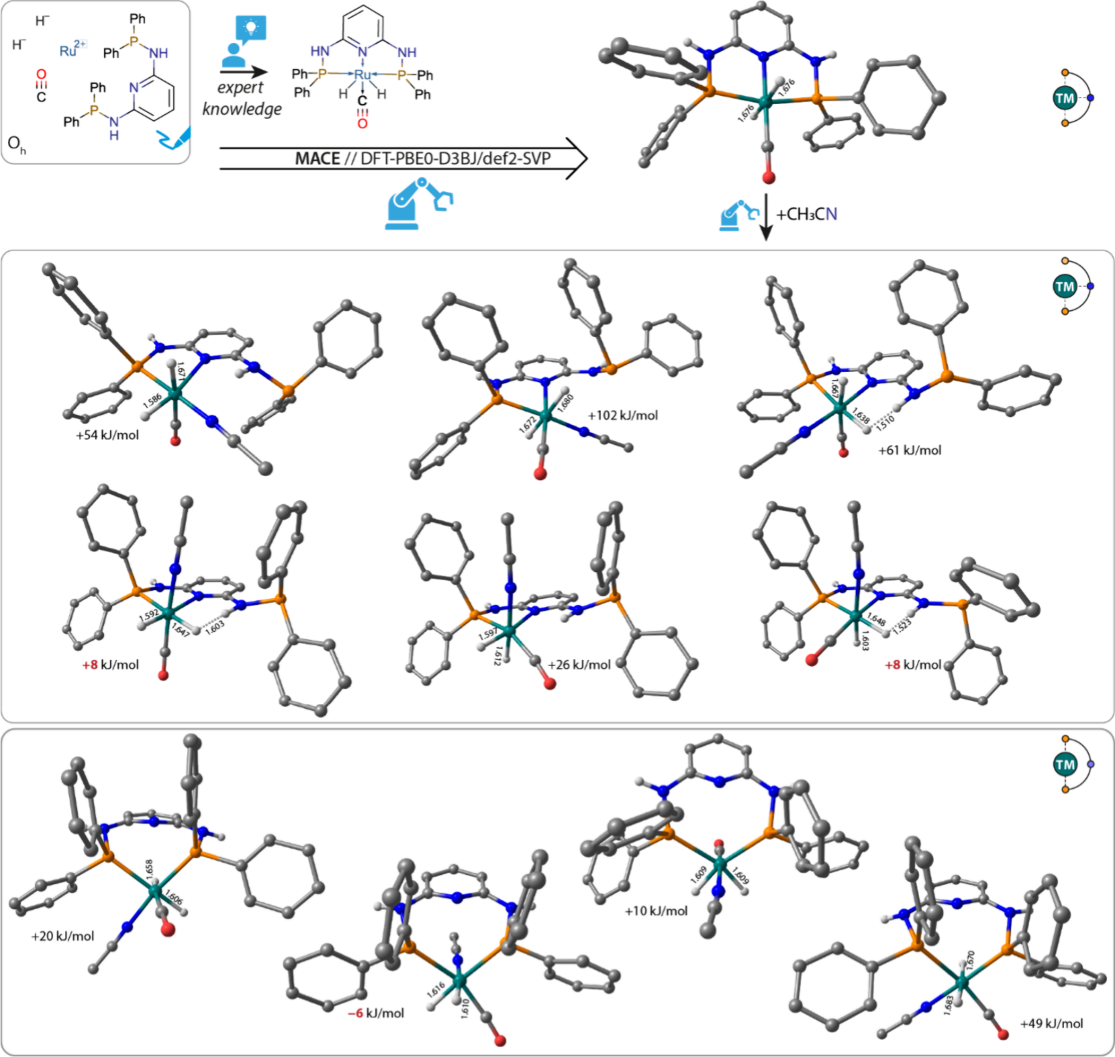
Fluxionality of the RuPN_3_P catalyst induced by MeCN coordination. Optimized structures of MACE-generated stereoisomers of the (top) octahedral RuH_2_CO(PN_3_P) complex and its adducts with MeCN formed through (middle) the P arm and (bottom) central N-donor decoordination. Energy values in kJ/mol represent the reaction energies (DE) for MeCN addition with most favorable coordinated modes given in red. Selected interatomic distances are given in Å. Hydrogens were omitted for clarity.

When all 3 donor heteroatoms of the ligand were specified in SMILES as the coordination sites, the protocol yielded an expected pincer geometry of the complex, featuring both hydride ions in the axial positions and equatorial CO ligand, in line with the experimental structural data (Figure 4). To study the principal possibility of the pincer donor hemilability in the presence of coordinating solvent, the starting definition of the complex components has been modified by introducing an additional auxiliary ligand MeCN (with the N center indicated as the coordinating donor) and specifying only 2 out of 3 donor centers on the PN_3_P pincer as the coordinating sites. Two situations were considered: (i) the hemilability of the phosphine pincer arms and (ii) decoordination of the central N donor atom of the pyridine backbone. The optimized geometries of the isomeric structures along with their relative stabilities expressed as the computed reaction energy (Δ*E*) for MeCN-induced ligand decoordination are summarized in Figure 4. The exchange of the P-arm with MeCN is unfavorable. Most of the structures featuring bidentate P,N-PN_3_P ligand generated by MACE are substantially higher (50–100 kJ/mol) in energy than those of the parent pincer complex. One specific ligand arrangement featuring cis-hydrides, equatorial CO, and axial MeCN ligands gave several less unfavorable configurations, which could be further stabilized by hydrogen bonding between the Ru-bound hydride and NH bridge of the tridentate ligand (Δ*E* = 8 kJ/mol).

To our surprise, more favorable MeCN coordination could be realized when decoordination of the pyridine N donor is considered. The resulting Ru-P,P-PN_3_P complexes (Figure 4, bottom) are characterized by much less unfavorable computed reaction energies, in general. One configuration featuring the same stable auxiliary ligand arrangement noted above resulted in a complex with a slightly exothermic reaction energy for MeCN coordination.

### High-Throughput Hemilability Exploration

**Figure 5:**
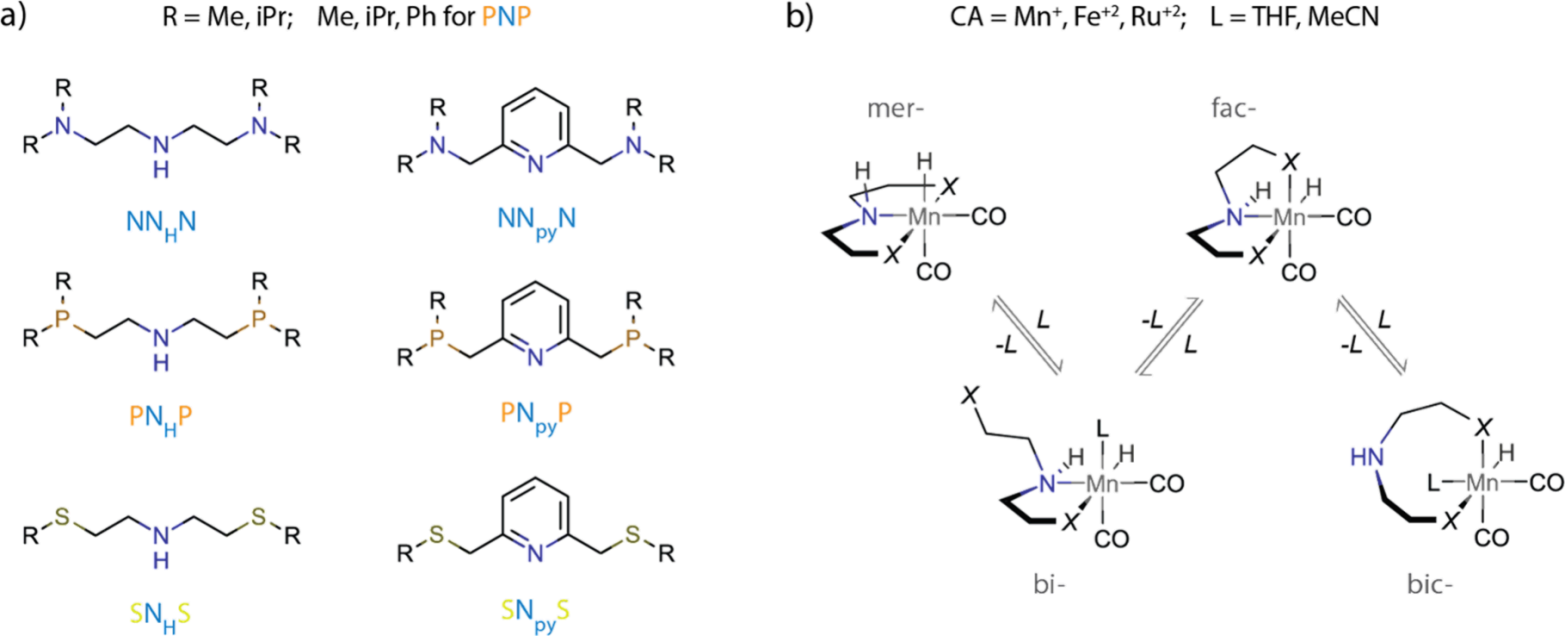
Explored chemical space of pincer ligands. (a) Structures of pincer ligands. (b) Considered configurations of pincers exemplified on Mn^+^ complexes and corresponding hemilabile equilibria.

Finally, to demonstrate the applicability of MACE as the first step in the high-throughput computational screening of coordination complexes, we decided to explore further the potential hemilability of a wider range of pincer complexes. We focused on studying the coordinational fluxionality of more flexible XNX-type pincer complexes of different transition metals. More specifically, we carried out an automated expert bias-free exploration of the relative stabilities of all possible coordination geometries for tri- and bidentate forms of pincer complexes and their dependence on the type of metal, ligand, and coordinating solvent. For this study, we considered the combinatorial set summarized in Figure 5a and featuring Mn^+^, Fe^2+^, and Ru^2+^ as the metal centers, NNN, PNP, and SNS-type ligands with the pyridine- or aliphatic amine central N donor fragment, and a representative set of substituents on the side arm X donors, namely, R = Me, ^i^Pr for -NR_2_ and -SR, and R = Me, ^i^Pr and Ph for -PR_2_. This combinatorial set was constructed to represent typical tridentate transition metal pincer complexes commonly considered in various reductive catalytic transformations such as (XNX)Mn(CO)_2_H, (XNX)Fe(CO)H_2_, and (XNX)Ru(CO)H_2_. The tridentate coordination is a common strategy allowing the stability of the catalyst to be enhanced, particularly when the transition to earth-abundant 3d metal-based systems is discussed.^36^ However, in the presence of coordinating solvents or intermediates, the formation of a bidentate configuration can be anticipated. Herein, we explore such a possibility by considering the decoordination of one of the donor atoms of the tridentate ligand and its substitution with a coordinating solvent molecule, namely, THF or MeCN (Figure 5b).

The highlight of our approach is that we do not assume any *a priori* preferred coordination of the tridentate ligand. The procedure generates all possible configurations such as the *fac*- and *mer*- for the tridentate binding mode, as well as *bi*- and *bic*- coordinations for the structures with donor hemilability (Figure 5b). Interestingly, such configurations are commonly assumed to be unstable and are omitted in reactivity analysis. In private conversation, many coordination chemistry and catalysis experts were particularly skeptical about the *bic*- configuration featuring the hemilabile central donor being among the lower-energy forms. The fallacy of the paths of expert knowledge can be further emphasized by the fact that after a three month break in the work for this paper, the first author forgot about the hypothesis on the existence of such a configuration, resulting in an extremely embarrassing conversation with the last author.

By using a simple Python script that is provided in the SI, we used MACE to generate all stereoisomers for each combination of metal, pincer, and solvent and the corresponding geometries, specifically 5 conformers for each stereoisomer. The obtained geometries were used as the starting points for DFT optimization at the PBE0-D3/6-31G(d,p)/SDD(Ru) level of theory. Afterward we specifically verified that the optimized geometry matched the starting configuration produced by MACE. The structures, for which the change in the connectivity has occurred, were discarded as the “high-energy”/“non-localizable” configurations (Figure S6). Finally, for each coordination type (*fac*-, *mer*-, *bi*-, and *bic*-) the lowest-energy configuration (stereoisomer and conformer) was selected, and their stabilities were assessed with respect to the respective benchmark *mer*-isomer.

**Figure 6:**
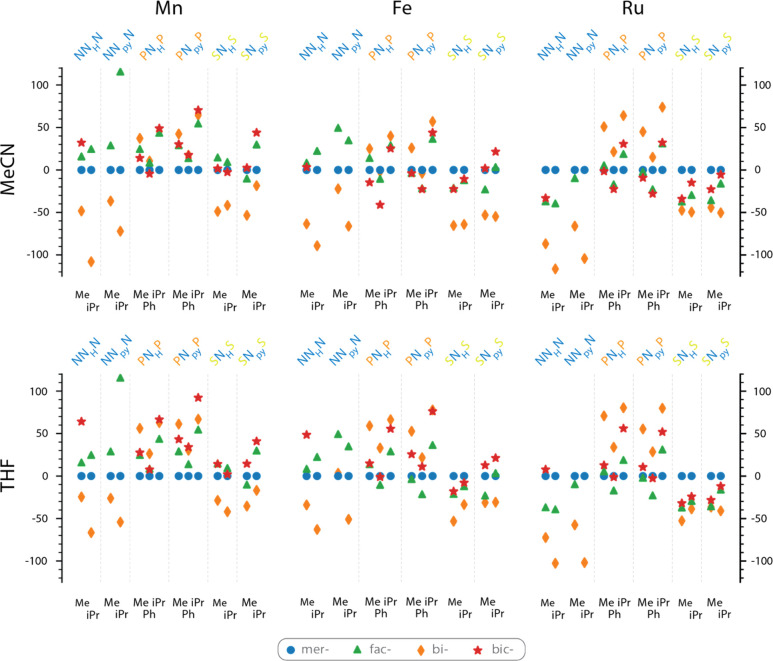
Relative stability of the most stable stereoisomers of explored metal complexes based on electronic energies obtained at the PBE0-D3/6-31G(d,p)/SDD(Ru) level of theory, kJ mol^–1^.

Figure 6 shows the dependence of the relative stabilties of *fac*-, *mer*-, *bi*-, and *bic*- configurations (designated with different symbols) on the central metal, the ligand backbone, and substituents at the donor atom, as well as the coordinating solvent. The relative stability is expressed as relative energies in kJ/mol with respect to the *mer*-configuration, taken as the 0 value for all systems.

The first feature that catches one’s eye is the pronounced preference for the formation of the *bi*- form for all complexes based on the NNN and SNS ligands with the respective energies being on average more than 50 kJ/mol lower than the corresponding PNP-based systems. Such a side arm hemilability is slightly more pronounced in the presence of a stronger coordinating MeCN solvent than THF, although the preference does not exceed 10 kJ mol^–1^. We assign this to the interplay of two main factors, namely, (i) the energy of the M–X bond and (ii) the bite angle of the pincer ligand. Indeed, because N and S are less strong donors than P, their substitution with a solvent molecule is more energetically favorable. The analysis of the optimized structures reveals that whereas the P–N bite angle in the PNP ligands for all systems is close to the optimal value of 90°, the respective N–N and S–N bite angles deviate from it and are, respectively, around 80° and 100°. The substitution of one of the pincer “arms” with a solvent molecule reduces the associated steric strain and stabilizes the *bi*- configuration.

The variations in stabilities of the *mer*- and *fac*- tridentate forms are less straightforward and seem to rely on the interplay of a wider range of factors. The nature of the central atom has a profound impact on the preference of the specific configuration. Our data reveal a pronounced preference for the formation of the *mer*- configuration for Mn and *fac*- for Ru, while their stabilities are comparable in the case of Fe complexes. The nature of the ligand backbone seems to have a less pronounced impact. We can note that the SNS ligands tend to stabilize the *fac*- form, and that for the Fe and Ru complexes with the NN_py_N ligand with ^i^Pr substituents the *fac*- form is destabilized by more than 100 kJ·mol^–1^ with respect to the *mer*-form. We assign this to the steric hindrance of two −N^i^Pr_2_ donor groups in adjacent positions of the complexes.

The most intriguing result of our analysis is the finding that some of the Fe complexes with rather conventional PNP ligands show a higher stability in the *bic*- form—the rather unexpected product of the central N atom hemilability. For several metal and ligand combinations, the *bic*-form either is the most stable or shows energy within 20 kJ/mol of the other most stable configuration. This implies that such hemilabile species may impact the reactivity and, for example, behavior of the catalyst systems. The stabilization of the *bic*- form relative to the *mer*- form is the highest with the relative energy of ca. −40 kJ/mol for the Fe complex of the PN_H_P, R = Ph ligand in the presence of MeCN. Apparently, for this system the highest ratio of the binding energy of the pincer arm and the binding energy of the central atom is achieved, which is compensated by the tangible energy of the Fe-NCMe bond, and the minimal steric hindrance as compared, for example, with the similar complex but R = ^i^Pr.

To support the last statement, we searched for the octahedral complexes of *bic*-coordinated XNX pincer ligands in the Cambridge Structural Database. The only complex that we found was DEMDUX, (XNX)Mo(CO)_4_ with XNX = Ph_2_P–N(Ph)–PPh_2_ (Figure S7). Its central donor atom has low donating ability due to conjugation with the phenyl group, which correlates with our hypothesis on the factors stabilizing the *bic*-form. DFT computations of possible configurations of this Mo complex supports our findings, predicting the relative energies of the possible forms as *bic*- (0 kJ/mol) < *bi*- (82 kJ/mol) < *fac*- (139 kJ/mol) < *mer*- (152 kJ/mol). The same results were obtained for the complex of the same ligand with the Fe(CO)H_2_ moiety and MeCN as an auxiliary ligand: *bic*- (0 kJ/mol) < *mer*- (89 kJ/mol) < *fac*- (91 kJ/mol) < *bi*- (108 kJ/mol).

## Conclusions

In summary, we present MACE—the software for automated exploration of stereoisomers of mononuclear octahedral and square-planar complexes and generation of their atomic coordinates suitable for quantum chemical studies. MACE copes well with complexes formed by multidentate ligands, failing rarely in the case of extremely sterically hindered systems. The versatility of the software is demonstrated in analyzing specific systems and as the starting point of high-throughput computational pipelines. MACE workflow and its detailed documentation are openly available via refs (24 and 29).

By employing MACE to analyze the hemilability of pincer ligands, we highlight the advantage of automation over expert knowledge and human factors. MACE revealed that in the examination of pincer complexes, it becomes imperative to account not only for the dissociation of the ligand arm but also for the dissociation of the central atom. The latter can be crucial, as it unveils that the stability of a particular configuration may hinge on *a priori* unknown factors, contributing to a more comprehensive understanding of the system.

## Supporting Information Available

Counting number of isomers for square planar and octahedral stereocenters, description of ligands extracted from CSD, and miscellaneous figures (PDF)
IPython notebooks comprehensively discussing all possible usage scenarios of the epic-mace package (ZIP)
Structures (XYZ) and energies (XLSX) of all metal complexes computed in this research and Python script used for the generation of starting geometries for the high-throughput computations (get_mace_geoms.py) (ZIP)
