# Supplementary material for: MACE: Automated Assessment of Stereochemistry of Transition Metal Complexes and Its Applications in Computational Catalysis
Source: J Chem Theory Comput. 2024 Feb 16;20(5):2313–20. doi: 10.1021/acs.jctc.3c01313 (PMC10938507; doi:10.1021/acs.jctc.3c01313)
Supplement: Supplementary file 1 — ct3c01313_si_001.pdf [file ct3c01313_si_001.pdf]

SUPPORTING INFORMATION FOR

MACE: Automated Assessment of Stereochemistry of Transition Metal Complexes and its Applications in Computational Catalysis

Ivan Yu. Chernyshov,[1,*] Evgeny A. Pidko[1,*]

[1] Inorganic Systems Engineering, Department of Chemical Engineering, Faculty of Applied Sciences, Delft University of Technology, Van der Maasweg 9, 2629 HZ, Delft, The Netherlands

* Corresponding authors: I.Chernyshov@TUDelft.nl, E.A.Pidko@TUDelft.nl

Counting number of isomers for square planar and octahedral stereocenters

The most thorough and clear justification of number of stereomers for square planar and octahedral stereocenters can be found in the OpenSMILES documentation [1], which we quote without changes below (reproduced from C.A. James, OpenSMILES specification, version 1.0, 2016-05-15, GNU Free Documentation License 1.2):

"3.8.5. Square Planar Centers

There are three tags to represent square planar stereochemistry: @SP1, @SP2 and @SP3. Since there is no way to determine to what chirality class an atom belongs based on the SMILES alone, the SP class is not the default class for tetravalent stereocenters. Therefore are the shorthand notations (@, @@) not equivalent to @SP1 and @SP2. That is, the full specification must be there (@SP followed by 1, 2 or 3). The square planar also differs from the other chiral primitives in that it does not use the notion of (anti-)clockwise. Instead, each primitive represents a shape that is formed by drawing a line starting from the atom that is first in the SMILES pattern to the next until the end atom is reached. This may result in 3 possible shaped which are referred to by a character with identical shape: 'U' for @SP1, '4' for @SP2 and 'Z' for @SP3. The graphical from of these shapes is illustrated in the image below.

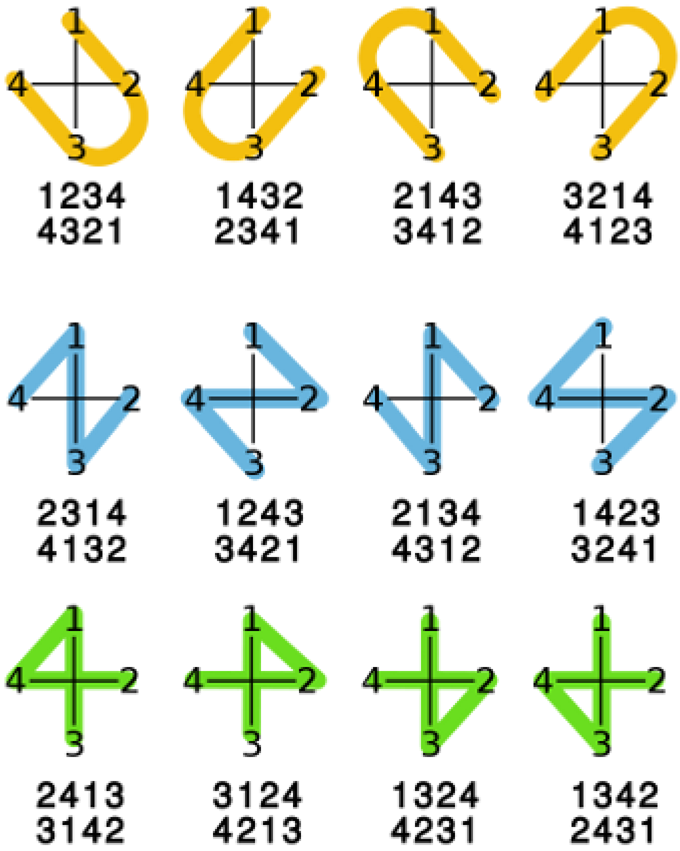

Figure S1. Shapes of chiral primitives of square planar centers.

3.8.7. Octahedral Centers

For 6 atoms, the unit permutation is (a, b, c, d, e, f). @OH1 means when viewing from a towards f, (b, c, d, e) are ordered anti-clockwise (@). @OH2 uses the same axis but the 4 intermediate atoms are ordered clockwise. The interpretation of the 28 remaining numbers is more complex though. The concept of shapes (see square planar stereochemistry) to describe the orientation of 4 atoms in a plane is reused. However, this time these shapes also have a clockwise or anti-clockwise winding. For the U shape, this is trivial since it means that the 4 atoms are listed clockwise or anti-clockwise. For the Z shape, the connection between the first two atoms determines the winding. Finally, for the 4 shape, the connection between the second and third atom determines the winding. The table below lists the shapes, axes and orders.

Table S1. Possible configurations of octahedral centers.

Shape	Viewing axis		OH number	Order
	From	Towards		
U	a	f	OH1	@
			OH2	@@
	a	e	OH3	@
			OH16	@@
	a	d	OH6	@
			OH18	@@
	a	c	19	@
			24	@@
	a	b	25	@
			30	@@
Z	a	f	4	@
			14	@@
	a	e	5	@
			15	@@
	a	d	7	@
			17	@@
	a	c	20	@
			23	@@

	a	b	26	@
			29	@@
4	a	f	10	@
			8	@@
	a	e	11	@
			9	@@
	a	d	13	@
			12	@@
	a	c	22	@
			21	@@
	a	b	28	@
			27	@@

”

[1] http://opensmiles.org/opensmiles.html, accessed on 10/01/2024

Description of ligands extracted from CSD

For a better understanding of the structure of ligands extracted from structural data on square planar and octahedral complexes we analyzed types of donor atoms, their counts, and their "topology" — connectivity of donor atoms in the molecule.

Statistics on donor atoms is provided in **Table S2**.

"Ligand topology" was defined as a simplified molecule containing donor atoms linked directly if they are neighbors (as in the coordinated peroxosolvate anion O_2^{2-}), or via dummy atom if there is a path between them in the molecular graph corresponding to the cycle formed by these two donor atoms and the central ion. This definition was found empirically and found effective for the representation and description of structure of complex multidentate ligands.

Statistics on "bare" topologies which does not contain info on types of donor atoms are provided in **Figures S2** and **S4** for square planar and octahedral ligands, respectively. The most popular topologies with info on donor atoms are provided in **Figures S3** and **S5** for square planar and octahedral ligands, respectively. In these figures, donor atoms are denoted by the "DA" label or the corresponding atomic symbol, atoms corresponding to linkers are denoted by the "*" symbol.

Table S2. Counts of various types of donor atoms in ligands extracted from CSD and used for the MACE-driven generation of square planar (SP) and octahedral (OH) complexes.

Donor atom	Hybridization	SP set	OH set
C	sp^2	155	106
N	sp	2	7
N	sp^2	568	3046
N	sp^3	271	443
P	sp^2	6	4
P	sp^3	295	129
As	sp^3	12	3
O	sp^2	37	351
O	sp^3	8	51
S	sp^2	41	9
S	sp^3	219	123
Se	sp^2	3	0
Se	sp^3	19	6

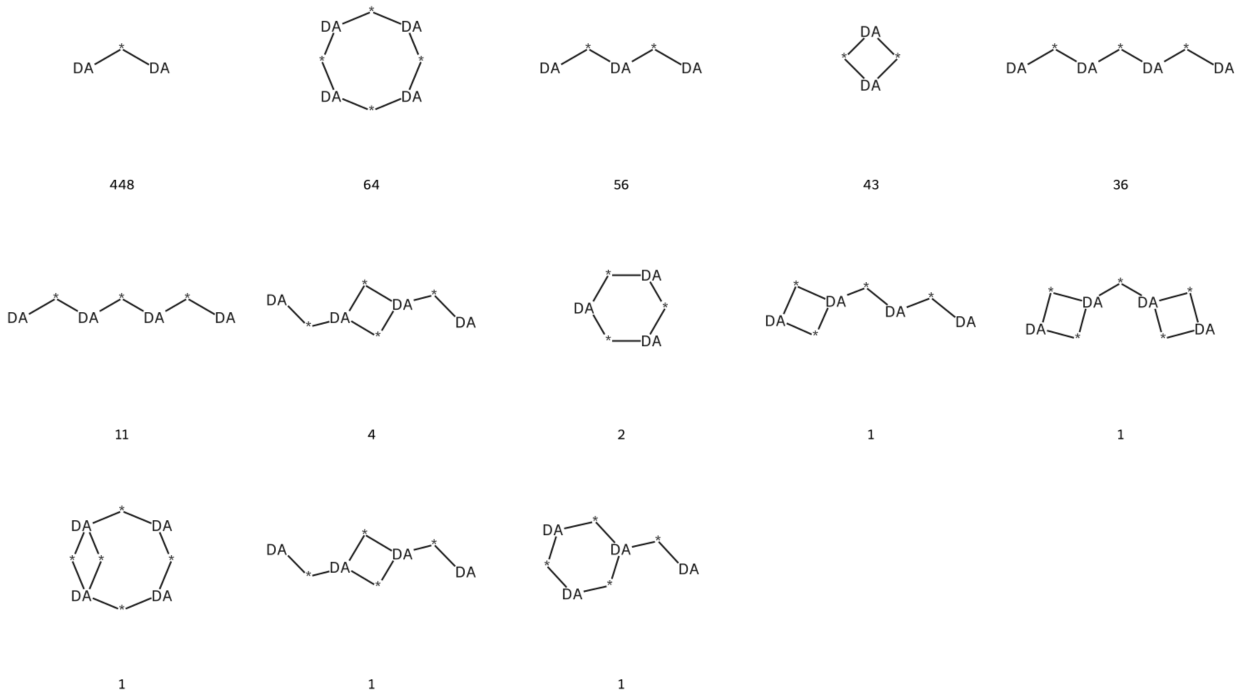

Figure S2. “Bare” topologies of ligands extracted from square planar complexes and their counts.

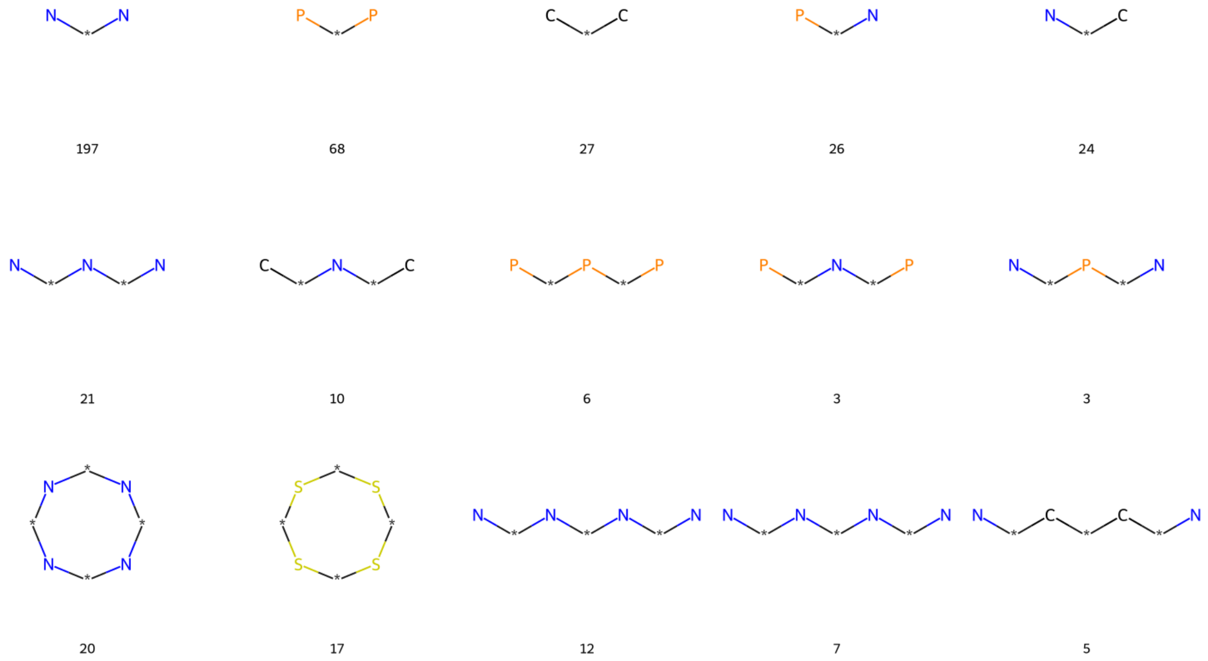

Figure S3. The most popular topologies of ligands with donor atoms extracted from square planar complexes for each denticity (2...4) and their counts.

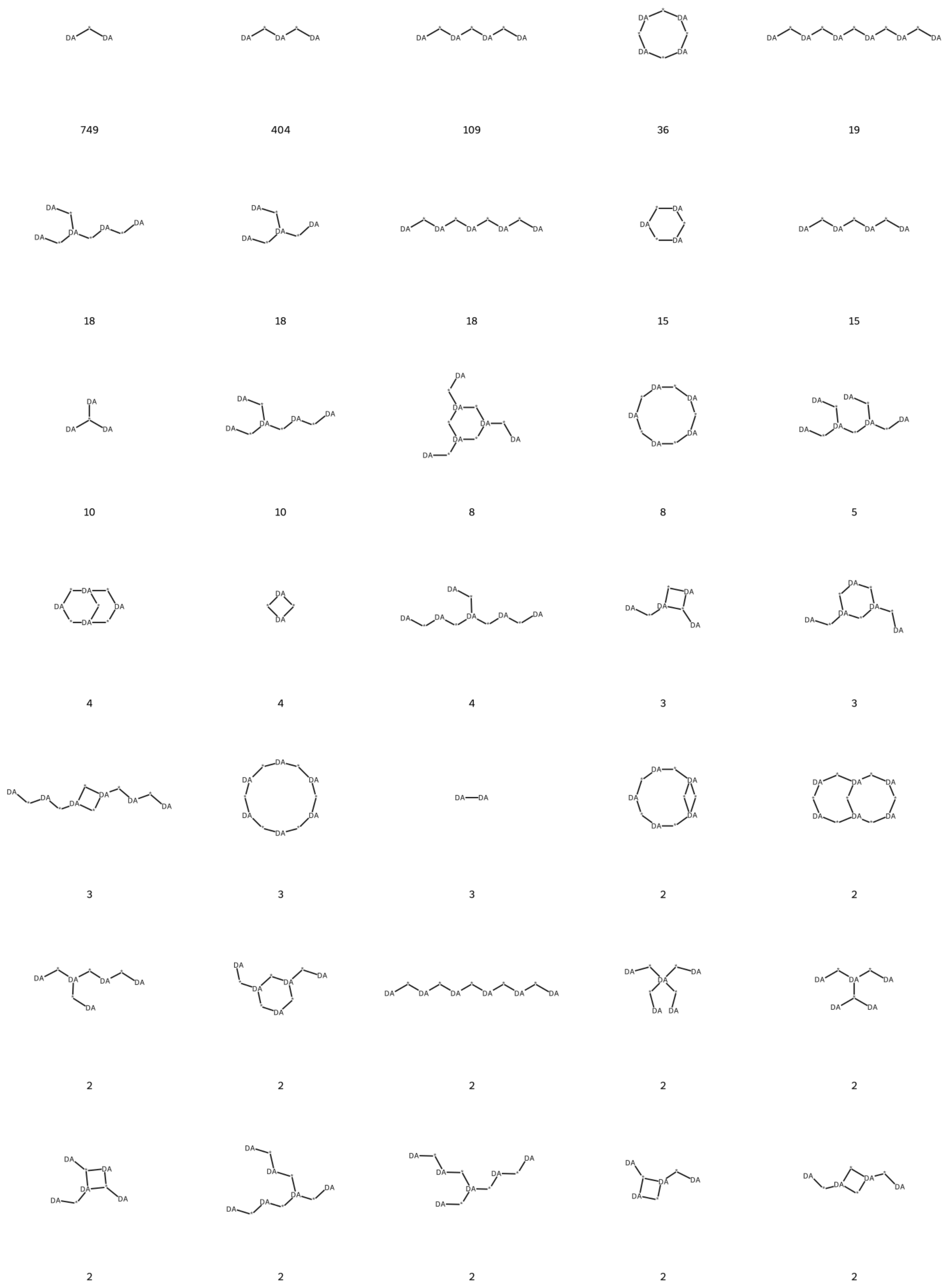
749
404
109
36
19
18
18
18
15
15
10
10
8
8
5
4
4
4
3
3
3
3
3
2
2
2
2
2
2
2
2
2
2
2
2

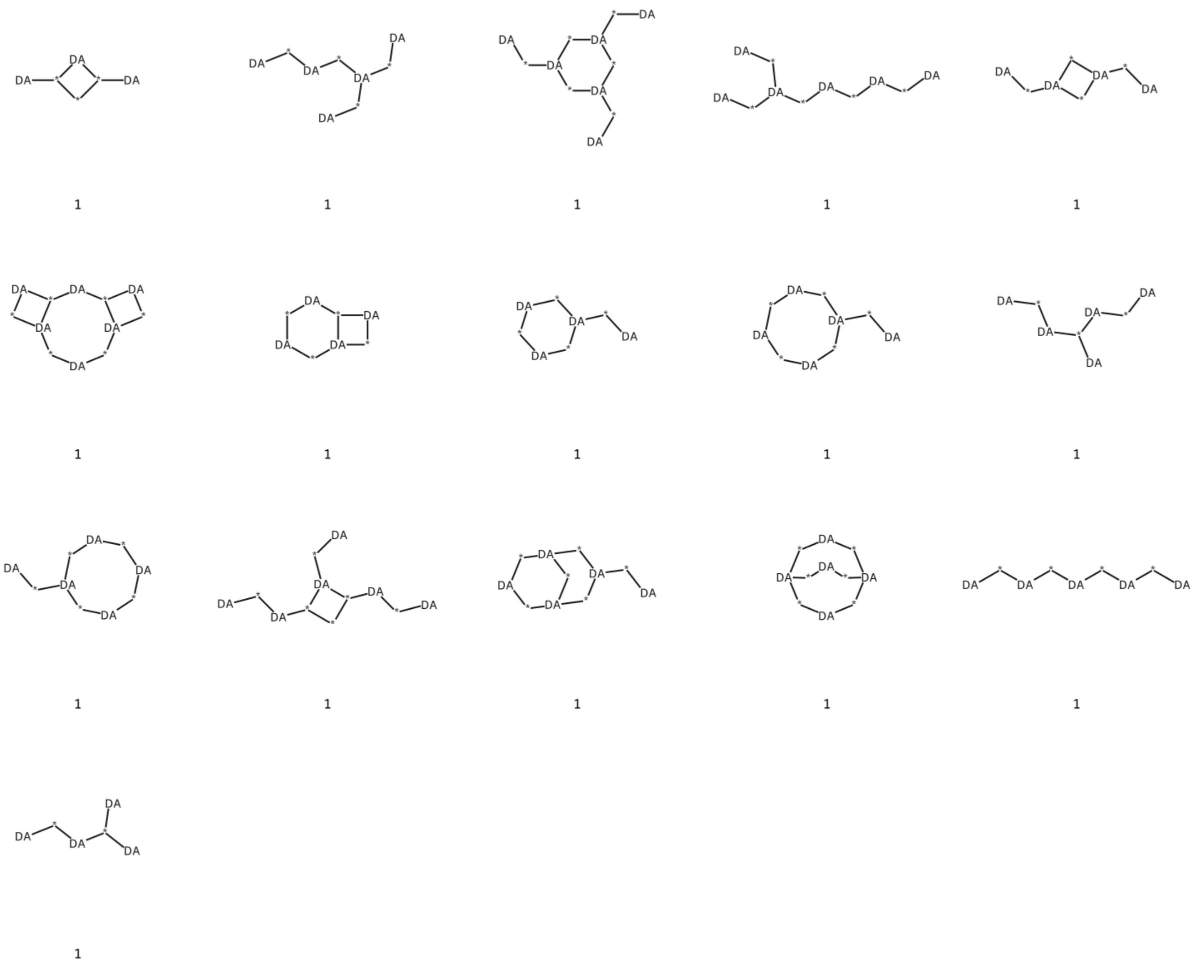

Figure S4. “Bare” topologies of ligands extracted from octahedral complexes and their counts.

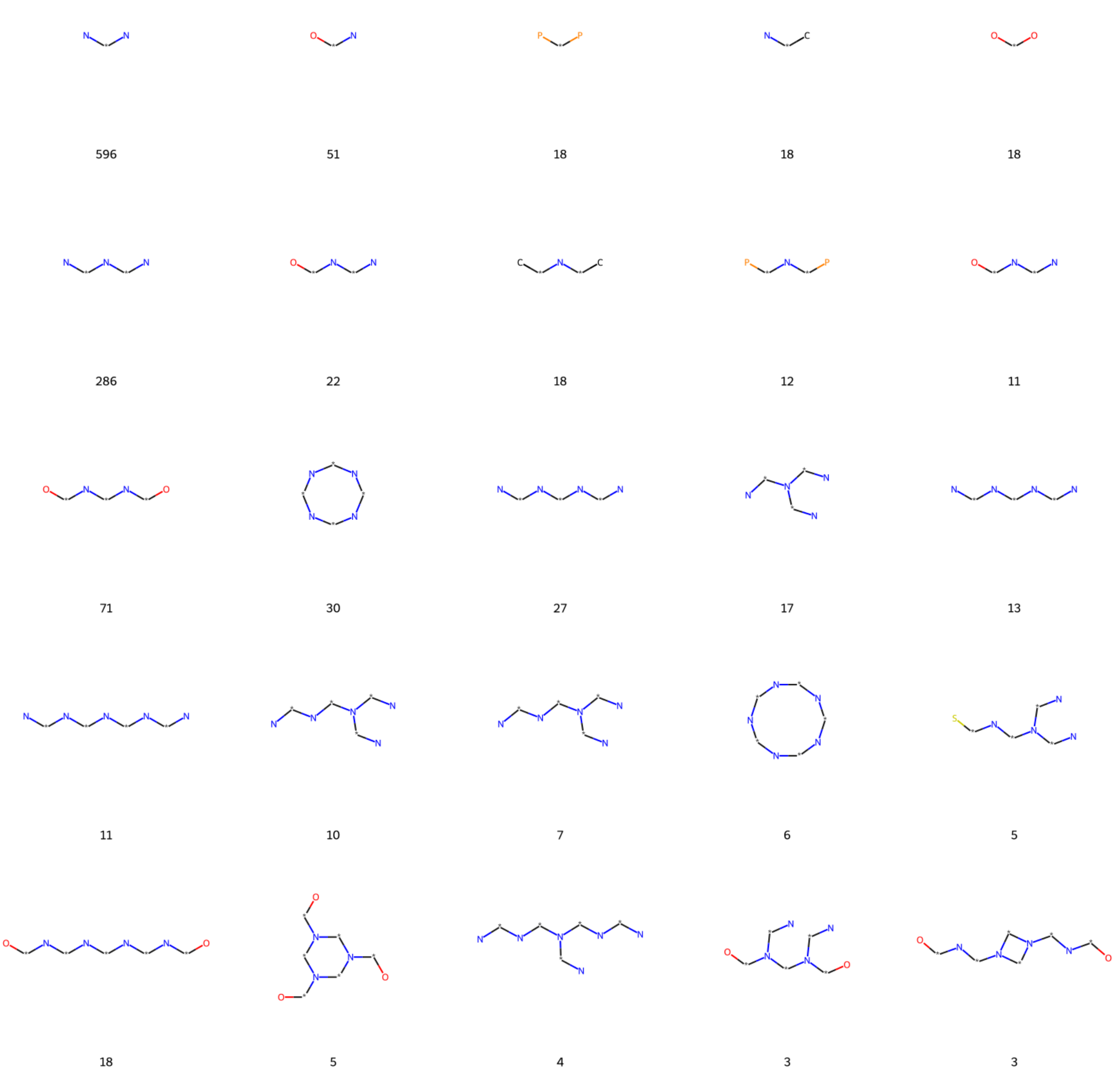

Figure S5. The most popular topologies of ligands with donor atoms extracted from octahedral complexes for each denticity (2...6) and their counts.

Miscellaneous

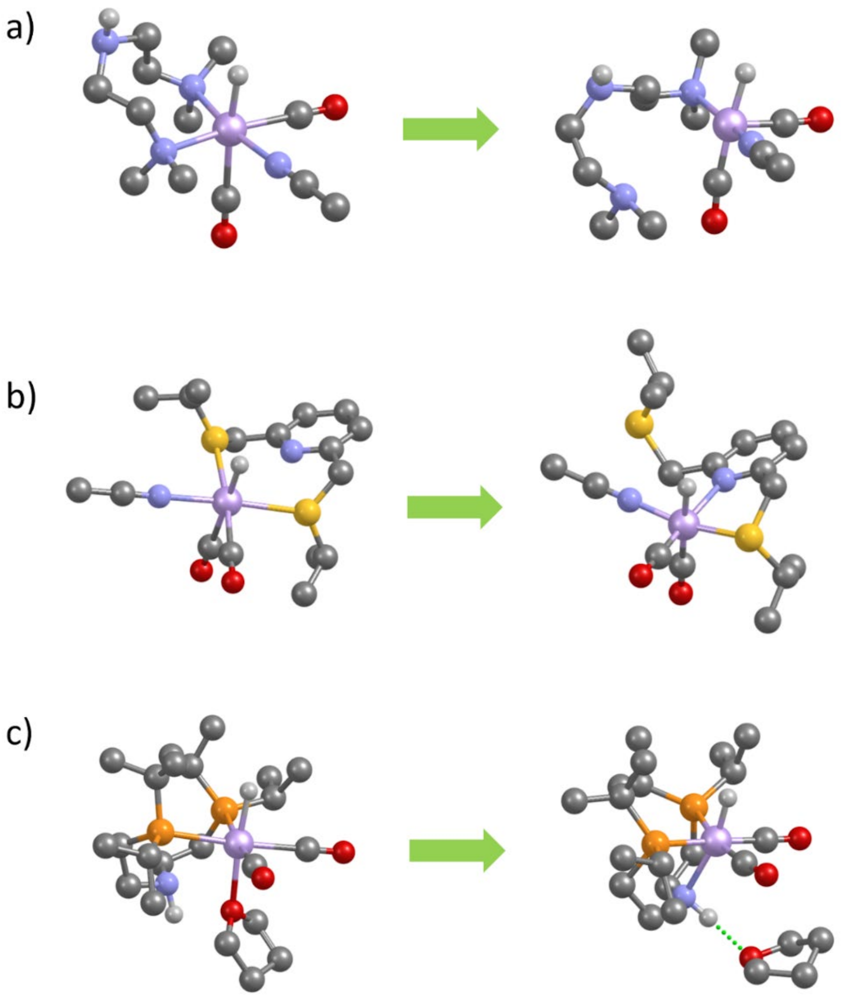

Figure S6. Representative examples of isomerization of transition metal complexes during the geometry optimization: (a) the "arm" bond dissociation; (b) *bic*- => *bi*- "isomerization"; (c) *bic*- => *fac*- "isomerization" with dissociation of an auxiliary ligand. Hydrogens were omitted for clarity.

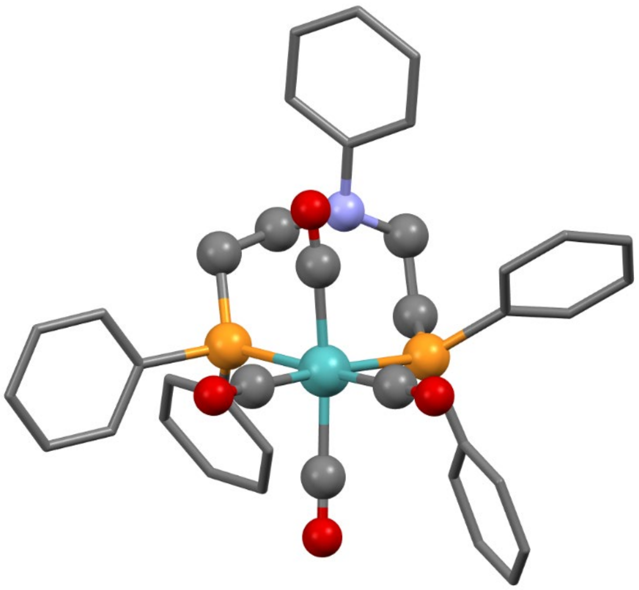

Figure S7. Structure of complex with the *bic*-coordinated PNP pincer (DEMDUX). Hydrogens were omitted for clarity.
